# The Welfare of Dogs and Cats in the European Union: A Gap Analysis of the Current Legal Framework

**DOI:** 10.3390/ani14172571

**Published:** 2024-09-04

**Authors:** Laura Contalbrigo, Simona Normando, Emma Bassan, Franco Mutinelli

**Affiliations:** 1 Companion Animal Welfare and Human–Animal Relationship Unit, National Reference Centre for Animal Assisted Intervention, Istituto Zooprofilattico Sperimentale delle Venezie, 35020 Legnaro, Italy; lcontalbrigo@izsvenezie.it (L.C.); fmutinelli@izsvenezie.it (F.M.); 2 Comparative Biomedicine and Animal Nutrition Department, University of Padua, 35020 Legnaro, Italy; simona.normando@unipd.it

**Keywords:** companion animal, Europe, breeding, legislation, wellbeing, pet

## Abstract

**Simple Summary:**

European citizens’ perception of dogs and cats has shifted, calling for a more robust and appropriate approach to protect the welfare of these pets. While recent updates to EU legislation have aimed to improve some aspects of companion animal welfare, these measures remain insufficient and lack harmonization across Member States. The existing legal framework still falls short of establishing a comprehensive, high standard of care and protection for these animals. Key issues include unhealthy and unethical breeding practices, irresponsible sale and ownership, the complexities of transporting animals for both commercial and non-commercial purposes, and inadequate regulations on training methods and working dog conditions. Additionally, there are concerns about the regulation of dog and cat shows, competitions, therapeutic neglect, end-of-life care, shelter management, and the rights of free-ranging cat and dog populations. To address these issues more effectively, there is a need for more consistent legislation across Europe, coupled with increased education and awareness of responsible pet ownership. The One Welfare approach, which emphasizes the interconnectedness of human and animal welfare, could also play a crucial role in bridging these legislative gaps, ensuring that the human–animal bond is better integrated into modern society’s welfare considerations. However, despite these efforts, many challenges remain unresolved, highlighting the need for further legislative action and societal engagement to fully protect companion animal welfare.

**Abstract:**

Companion animals, especially dogs and cats, have increasingly been recognized as moral subjects and valued as family members by European citizens. This new role encourages policy makers to face the many companion animals’ welfare issues not yet covered by the EU legislation. The main gaps in the protection of dog and cat welfare during their all lifespan have been identified: unhealthy and unethical breeding practices, irresponsible sale and ownership, transport for commercial and non-commercial purposes, training methods, working dog conditions and rights, regulation of dog and cat shows and competitions, the therapeutic neglect, dog and cat end-of-life care, shelter management legislation and the free-ranging cat and dog population rights. The EU legislation framework is still very weak and far from establishing a harmonized approach, promoting a high standard of care and protection across Member States. We conclude that education and awareness regarding responsible pet ownership and the need for a One Welfare approach have a high value in finding adequate solutions, especially when poor human social welfare affects companion animal welfare. Given the link between human and companion animal welfare, the use of stakeholders’ involvement strategies and a transdisciplinary approach appear crucial for the development of an EU legal framework for the well-being of dogs and cats.

## 1. The Legal and Socio-Economic Framework

Over the last 50 years, companion animals, especially dogs and cats, have gained a new position in European society: they are moral subjects and are considered family members by many European citizens [1]. In 2022, approximately 106 million dogs and 129 million cats lived in EU citizens’ households [2]. It is estimated that in the EU between 24,000 and 30,000 commercial dog breeders and between 8000 and 10,000 commercial cat breeders work with a trade market volume of 1.3 billion per year [3]. Only 20% of them are pedigree breeders. A total of 60% of the EU market for dogs and cats is developed online [3].

In 2023, the Eurobarometer highlighted that 44% of European citizens own companion animals and almost 74% of respondents support the need for rules to have better protection for the welfare of pet animals in their country [4]. Furthermore, over eight in ten European respondents consider that breeding practices should meet basic ethical requirements for all domestic animals including pets [4]. The new social role of pets and the market volume of their trade encourage policy makers to face the many companion animals’ welfare issues not yet covered by the EU legislation [5]. 

Since 1987, some provisions to establish standards of attitude and practice towards pet ownership, breeding, and pet trades have been provided through the European Convention for the Protection of Pet Animals (ETS N. 125). In 1995, the Convention was followed by some Resolutions aimed at harmonizing the implementation of the Convention by Member States (e.g., Resolution on surgical operations in pet animals; Resolution on the breeding of pet animals; Resolution on the keeping of wild animals as pets).

Moreover, some additional actions were taken that directly or indirectly consider the welfare of pets, but with many limitations:

- Council Regulation (EC) N. 1/2005 of 22 December 2004 on the protection of animals during transport and related operations and amending Directives 64/432/EEC and 93/119/EC and Regulation (EC) No. 1255/97;
- Regulation (EC) No. 1523/2007 of the European Parliament and of the Council of 11 December 2007 banning the placing on the market and the import to, or export from, the Community of cat and dog fur, and products containing such fur;
- Directive 2010/63/EU of the European Parliament and of the Council which regulates the keeping, breeding, and supply of animals kept for scientific purposes (including dogs and cats);
- Regulation (EU) No. 576/2013 of the European Parliament and of the Council of 12 June 2013 on the non-commercial movement of pet animals and repealing Regulation (EC) No 998/2003;
- Commission implementing Regulation (EU) No. 577/2013 of 28 June 2013 on the model identification documents for the non-commercial movement of dogs, cats, and ferrets; the establishment of lists of territories and third countries; and the format, layout, and language requirements of the declarations attesting compliance with certain conditions provided for in Regulation (EU) No. 576/2013 of the European Parliament and of the Council.

Indeed, all these legislative acts are addressed to protect dogs and cats in a specific context, for example, when used for scientific purposes or during commercial and non-commercial transport. They do not consider the welfare of these animals in the industry focusing on the health and welfare issues resulting from the breeding, keeping, trading, and supplying of dogs and cats in the EU [6,7]. 

Even in the two EU regulations that have recently changed the legal framework of animal health, food and feed production, plant health, and plant protection products in Europe, cats and dogs are mentioned. Regulation (EU) 2016/429 of the European Parliament and of the Council of 9 March 2016 on transmissible animal diseases and amending and repealing certain acts in the area of animal health (‘Animal Health Law’) classifies dogs and cats as pets in Annex I. In this Annex, the European legislator listed the species considered “companion animals” in Europe by law, going beyond the scientific and ethical discussion about this animal category [8]. 

The regulation covers their movements between Member States and from third countries in the context of animal diseases (most notably rabies) and requires the identification of dogs and cats with a transponder. However, the Regulation does not establish precise standards about transponders, registrations, databases used, and their interoperability and complete pet traceability.

In the Regulation (EU) 2017/625 of the European Parliament and of the Council of 15 March 2017 on official controls and other official activities performed to ensure the application of food and feed law, rules on animal health and welfare, plant health, and plant protection products, the Commission approved a single legislative framework for the organization of official controls. This framework will fit also for the breeding and trade of cats and dogs as soon as requirements are established. Indeed, the absence of an EU regulation for breeding, keeping, and placing on the market of dogs and cats as well as the lack of harmonization among national rules of some European countries (where existing) often cause circumstances detrimental to the welfare of dogs and cats. Welfare problems concerning dogs and cats can affect both physical and behavioral aspects [9]. Some breed standards and selection practices favor inherited disease diffusion [10] in the populations of pedigree dogs and cats [11]. The poor sanitary management of breeding stocks and stress during transport and commercialization can lead to many health issues spreading high-risk infectious diseases [12] and zoonosis. Moreover, early-life and parental experiences are potential contributors to the developmental origins of health and disease (DOHaD) in adulthood [13]. 

Even the main behavioral problems like generalized anxiety disorders, separation anxiety, and phobia in dogs [14] and complaints like house soiling in dogs and cats can originate from poor management conditions at the kennel or at the cattery [15,16,17].

## 2. The New Regulation Proposal (2023/0447)

In December 2023, a proposal for a Regulation of the European Parliament and of the Council on the welfare of dogs and cats and their traceability was published. At its meeting on 26 June 2024, Coreper approved the revised Presidency compromise text on the above-mentioned proposal and agreed that, according to this text, negotiations could start with the European Parliament in the context of the ordinary legislative procedure.

This regulation aims to protect the welfare of cats and dogs according to the “Five Domains Model” used to describe the different dimensions of animal welfare. This model does not focus only on the absence of negative experiences for the animal but also encompasses positive experiences [18].

This proposal shall be applied to the breeding, keeping, trading, and supplying of dogs and cats with or without pedigree as pets in the EU establishing minimum requirements for the following:

- The welfare of dogs and cats bred or kept in establishments or placed on the EU market;
- The traceability of dogs and cats placed on the EU market or supplied in the EU countries.

Therefore, this regulation proposal considers the following:

- Traceability of dogs and cats bred in Europe: transponder implantation, registrations of animals, and breeding centers;
- Traceability of dogs and cats coming from third countries: same welfare and identification standards of pets bred and raised outside the EU;
- Regulation of online advertisements;
- Common welfare standards: requirements for housing, breeding, and care of dogs and cats;
- Responsible ownership: future owners must be aware of proper pet care, veterinary needs, and nutrition;
- Competences of animal caretakers: breeding centers, pet shops, and shelters must have animal caretakers with proper knowledge, skills, and competencies in the care and welfare of housed animals.

This Regulation can reduce all risks related to poor traceability like public health and animal health risks (zoonotic diseases spread, animal diseases diffusion, poor breeding, and animal care), unfair competition among commercial breeders and pet shops, and all risks for consumers (frauds, animals with health and behavioral problems).

The Regulation should be followed by some implementations already well known:

- Harmonization of the education, training, and professional experience of animal caretakers;
- Identification of information to be provided by suppliers as proof of identification and registration of dogs and cats (both online and by other means);
- Details about check systems to verify the authenticity of the identification and registration of dogs and cats;
- Minimum requirements for databases and their interoperability;
- Harmonization of the methodology for collecting and measuring animal welfare data and the reporting of these data by Member States to the Commission;
- Procedures to recognize the equivalence to EU requirements of dogs and cats bred and kept in third countries, which intend to import animals to the EU with specific provisions regarding establishments.

## 3. The Gap Analysis 

The Regulation proposal helps to go through the development of better conditions for the breeding and keeping of dogs and cats in the EU. However, some additional points need to be considered and management strategies need to be implemented by the EU:

1. Unhealthy and unethical breeding practices that push towards hyper-types or suffering phenotypes: unhealthy dog and cat breeding practices should be considered genetic abuse [11,19]. Thanks to the development of epidemiological studies and DNA technologies, scientific evidence about genetic disorders and their detrimental consequences on dog and cat health and welfare at both individual and population levels forced the adoption of new approaches to dog and cat breeding programs, highlighting the need of a stronger leadership of kennel and cattery clubs in mentoring breeders to address their breeding programs;
2. Actions to contrast irresponsible sales and ownership [20,21]. This should include actions to improve citizen culture about pet care and needs, to reduce neglect, therapeutic neglect [22], and other social phenomena involving pets like hoarding, dog aggressions, and pet abuse [23]. These actions should be developed through a transdisciplinary approach and at different levels in society starting with children’s education for a respectful and positive human–animal relationship [24,25];
3. Dog training methods and dog trainers’ education, competencies, responsibilities, and registration as professionals. Dog trainers have an important social role in the management of dog behavioral problems and in the education of owners for a positive human-dog relationship. Nowadays, we have no harmonization of the competencies and responsibilities of these professionals in Europe even if scientific literature stresses the impact of training methods on dog welfare [26,27];
4. Working dog conditions and rights (including assistance dogs) with the development of guidelines for dog welfare requirements in the industry, along the supply chain. Dogs are involved in many kinds of work on behalf of, for, and with people in strong cooperation with their handlers: their work should be founded on respect, reciprocity, and protection of rights [28]. In this perspective, new insight into interspecies justice can be investigated highlighting how animal work needs to be addressed for animal self-realization, skills development and pleasure, autonomy, and agency [28];
5. Shared rules for dog shows and competitions and cat shows can ensure the health and welfare of the animals involved [29,30]. Dog and cat shows are not only the celebration of beauty, attitudes, good genetic selection, and love for our pets. They are often contexts where extreme conformational traits and appearance are glorified, rather than the health, comfort, and welfare of the animals, which are secondary considerations, resulting in neglect of their basic needs [31].
6. Free-ranging cat and dog population rights (e.g., rules for their management and protection as well as governance strategies for their reductions);
7. A complete shelter management legislation that includes the necessary professional figures required in shelter staff, as well as delineates organizational and operational procedures, including the implementation of a transparent financial management system. Given that animal shelters are transitional spaces for dogs and cats, one of the priorities for volunteers and staff should be the re-homing process optimizing the match between animals and adopting families [32]. This approach aims to reduce the risk of abandonment or the return of animals following unsuccessful adoptions. To achieve this, the adoption team should receive proper training and comprehensive “adoption guidelines” should be developed based on scientific evidence [33]. These guidelines should include selection criteria for prospective adopters based on both psychological and demographic factors, as well as general criteria for adopter-animal matching that can be tailored to individual cases. This will ensure impartiality and fairness during the assignment of animals to new families, thereby facilitating optimal re-homing outcomes [34]. Additionally, the legislation should outline the inspections that the competent authority is obligated to conduct, along with the data that shelters must provide to the competent authority, including financial information;
8. Legislative actions addressed to support the human–animal bond for all European citizens including the more vulnerable members of the EU society, e.g., elderly people with a social impairment or mental diseases [35]. The need for pet-friendly service networks and a community care approach that considers not only people but also their loved pets opens new perspectives and frontiers in social welfare policy programs and in the emergency management guidelines of the EU that are necessary to guarantee both pet and people’s well-being.

## 4. Conclusions

In conclusion, the education and awareness regarding responsible pet ownership and the need for a One Welfare [36] approach hold significant value in identifying adequate solutions to problems affecting companion animal welfare. Given the link between human and companion animal welfare, the implementation of stakeholders’ involvement strategies and a transdisciplinary approach appear crucial for the development of an EU legal framework aimed at promoting the well-being of companion animals.

## Data Availability

No new data were created or analyzed in this study.

## References

[1] Morais R.C. Dog Days. https://www.forbes.com/forbes/2004/0621/078.html.

[2] (2024). FEDIAF Annual Report. https://europeanpetfood.org/wp-content/uploads/2024/06/FEDIAF-Annual-Review-2024_Online.pdf.

[3] European Commission (2023). Proposal for a Regulation of the European Parliament and of the Council on the Welfare of Dogs and Cats and Their Traceability. https://food.ec.europa.eu/system/files/2023-12/aw_reg-proposal_2023-769_dog-cat-trace.pdf.

[4] European Commission (2023). Attitudes of Europeans towards Animal Welfare. https://europa.eu/eurobarometer/surveys/detail/2996.

[5] European Commission Platform Conclusions. https://food.ec.europa.eu/animals/animal-welfare/eu-platform-animal-welfare/platform-conclusions_en.

[6] Cardoso S.D., Faraco C.B., de Sousa L., Da Graça Pereira G. (2017). History and Evolution of the European Legislation on Welfare and Protection of Companion Animals. J. Vet. Behav..

[7] Fossati P., Ruffo G. (2021). Purebred Dogs and Cats: A Proposal for a Better Protection. J. Vet. Behav..

[8] Pongrácz P., Dobos P. (2023). What Is a Companion Animal? An Ethological Approach Based on Tinbergen’s Four Questions. Critical Review. Appl. Anim. Behav. Sci..

[9] Sonntag Q., Overall K.L. (2014). Key Determinants of Dog and Cat Welfare: Behaviour, Breeding and Household Lifestyle. Rev. Sci. Tech. l’OIE.

[10] McGreevy P. (2007). Breeding for Quality of Life. Anim. Welf..

[11] Morel E., Malineau L., Venet C., Gaillard V., Péron F. (2024). Prioritization of Appearance over Health and Temperament Is Detrimental to the Welfare of Purebred Dogs and Cats. Animals.

[12] Anderson M.E.C., Stull J.W., Weese J.S. (2019). Impact of Dog Transport on High-Risk Infectious Diseases. Vet. Clin. North Am. Small Anim. Pract..

[13] Gaillard V., Chastant S., England G., Forman O., German A.J., Suchodolski J.S., Villaverde C., Chavatte-Palmer P., Péron F. (2022). Environmental Risk Factors in Puppies and Kittens for Developing Chronic Disorders in Adulthood: A Call for Research on Developmental Programming. Front. Vet. Sci..

[14] Tiira K., Lohi H. (2015). Early Life Experiences and Exercise Associate with Canine Anxieties. PLoS ONE.

[15] Dietz L., Arnold A.-M.K., Goerlich-Jansson V.C., Vinke C.M. (2018). The Importance of Early Life Experiences for the Development of Behavioural Disorders in Domestic Dogs. Behaviour.

[16] Buttner A.P., Awalt S.L., Strasser R. (2023). Early Life Adversity in Dogs Produces Altered Physiological and Behavioral Responses during a Social Stress-buffering Paradigm. J. Exp. Anal. Behav..

[17] Finka L.R. (2022). Conspecific and Human Sociality in the Domestic Cat: Consideration of Proximate Mechanisms, Human Selection and Implications for Cat Welfare. Animals.

[18] Mellor D.J., Beausoleil N.J., Littlewood K.E., McLean A.N., McGreevy P.D., Jones B., Wilkins C. (2020). The 2020 Five Domains Model: Including Human–Animal Interactions in Assessments of Animal Welfare. Animals.

[19] Menor-Campos D.J. (2024). Ethical Concerns about Fashionable Dog Breeding. Animals.

[20] Rioja-Lang F., Bacon H., Connor M., Dwyer C.M. (2019). Determining Priority Welfare Issues for Cats in the United Kingdom Using Expert Consensus. Vet. Rec. Open.

[21] Glanville C.R., Hemsworth L.M., Hemsworth P.H., Coleman G.J. (2023). Duty of Care in Companion Dog Owners: Preliminary Scale Development and Empirical Exploration. PLoS ONE.

[22] Normando S., De Santis M., Mingardo L., Contalbrigo L. Animal therapeutic neglect: When does it mean animal abuse?. Proceedings of the 5th Annual Meeting of the European Veterinary Congress of Behavioural Medicine and Animal Welfare.

[23] van Herwijnen I.R., van der Borg J.A.M., Kapteijn C.M., Arndt S.S., Vinke C.M. (2023). Factors Regarding the Dog Owner’s Household Situation, Antisocial Behaviours, Animal Views and Animal Treatment in a Population of Dogs Confiscated after Biting Humans and/ or Other Animals. PLoS ONE.

[24] Ngai J.T.K., Yu R.W.M., Chau K.K.Y., Wong P.W.C. (2021). Effectiveness of a School-Based Programme of Animal-Assisted Humane Education in Hong Kong for the Promotion of Social and Emotional Learning: A Quasi-Experimental Pilot Study. PLoS ONE.

[25] Tardif-Williams C.Y., Bosacki S.L. (2015). Evaluating the Impact of a Humane Education Summer-Camp Program on School-Aged Children’s Relationships with Companion Animals. Anthrozoos.

[26] Vieira de Castro A.C., Fuchs D., Morello G.M., Pastur S., de Sousa L., Olsson I.A.S. (2020). Does Training Method Matter? Evidence for the Negative Impact of Aversive-Based Methods on Companion Dog Welfare. PLoS ONE.

[27] Casey R.A., Naj-Oleari M., Campbell S., Mendl M., Blackwell E.J. (2021). Dogs Are More Pessimistic If Their Owners Use Two or More Aversive Training Methods. Sci. Rep..

[28] Blattner C.E., Coulter K., Kymlicka W. (2019). Animal Labour.

[29] Hurley S. (2018). Dog Showing and Training: Enjoyable Hobbies or Destructive Practices That Reinforce Speciesist Ideologies?. Domestic Animals, Humans, and Leisure: Rights, Welfare, and Wellbeing.

[30] Georgie B. (2016). Celebrations—And Controversy—At the 125th Crufts Dog Show. Vet. Rec..

[31] Woods V., Dean R., Kuhl C., Lea R. (2016). Pedigree Dog Showing in the UK: Show Exhibitors Attitudes towards Pedigree Dog Health. BSAVA Congress Proceedings 2016.

[32] Koralesky K.E., Rankin J.M., Fraser D. (2023). Animal Sheltering: A Scoping Literature Review Grounded in Institutional Ethnography. Anim. Welf..

[33] Griffin K.E. (2024). The Application of Scientific Evidence-Based Changes to an Animal Shelter’s Rehoming Practices. Anim. Behav. Welf. Cases.

[34] Stavisky J., Brennan M.L., Downes M.J., Dean R.S. (2017). Opinions of UK Rescue Shelter and Rehoming Center Workers on the Problems Facing Their Industry. Anthrozoos.

[35] Applebaum J.W., MacLean E.L., McDonald S.E. (2021). Love, Fear, and the Human-Animal Bond: On Adversity and Multispecies Relationships. Compr. Psychoneuroendocrinol..

[36] Pinillos R.G. (2018). One Welfare: A Framework to Improve Animal Welfare and Human Well-Being.

